# Electrically-evoked oscillating calcium transients in mono- and co-cultures of iPSC glia and sensory neurons

**DOI:** 10.3389/fncel.2023.1094070

**Published:** 2023-03-16

**Authors:** Jennifer Lawson, Elijah LaVancher, Mauricio DeAlmeida, Bryan James Black

**Affiliations:** Department of Biomedical Engineering, Francis College of Engineering, University of Massachusetts Lowell, Lowell, MA, United States

**Keywords:** induced pluriopotent stem cells, microelectrode array (MEA), calcium transient, nociception and pain, phenotypic assay

## Abstract

Activated glia are known to exhibit either neuroprotective or neurodegenerative effects, depending on their phenotype, while participating in chronic pain regulation. Until recently, it has been believed that satellite glial cells and astrocytes are electrically slight and process stimuli only through intracellular calcium flux that triggers downstream signaling mechanisms. Though glia do not exhibit action potentials, they do express both voltage- and ligand-gated ion channels that facilitate measurable calcium transients, a measure of their own phenotypic excitability, and support and modulate sensory neuron excitability through ion buffering and secretion of excitatory or inhibitory neuropeptides (i.e., paracrine signaling). We recently developed a model of acute and chronic nociception using co-cultures of iPSC sensory neurons (SN) and spinal astrocytes on microelectrode arrays (MEAs). Until recently, only neuronal extracellular activity has been recorded using MEAs with a high signal-to-noise ratio and in a non-invasive manner. Unfortunately, this method has limited compatibility with simultaneous calcium transient imaging techniques, which is the most common method for monitoring the phenotypic activity of astrocytes. Moreover, both dye-based and genetically encoded calcium indicator imaging rely on calcium chelation, affecting the culture’s long-term physiology. Therefore, it would be ideal to allow continuous and simultaneous direct phenotypic monitoring of both SNs and astrocytes in a high-to-moderate throughput non-invasive manner and would significantly advance the field of electrophysiology. Here, we characterize astrocytic oscillating calcium transients (OCa^2+^Ts) in mono- and co-cultures of iPSC astrocytes as well as iPSC SN-astrocyte co-cultures on 48 well plate MEAs. We demonstrate that astrocytes exhibit OCa^2+^Ts in an electrical stimulus amplitude- and duration-dependent manner. We show that OCa^2+^Ts can be pharmacologically inhibited with the gap junction antagonist, carbenoxolone (100 μM). Most importantly, we demonstrate that both neurons and glia can be phenotypically characterized in real time, repeatedly, over the duration of the culture. In total, our findings suggest that calcium transients in glial populations may serve as a stand-alone or supplemental screening technique for identifying potential analgesics or compounds targeting other glia-mediated pathologies.

## Introduction

Neurons, along with associated glia of the peripheral and central nervous systems (e.g., Schwann cells, satellite glial cells, astrocytes, and oligodendrocytes), comprise what may be considered a fundamental functional neuroglial unit (De Luca et al., 2020). Through bidirectional electrochemical (Ahmadpour et al., 2021; Benfey et al., 2021; McNeill et al., 2021) and paracrine signaling (Rose et al., 2017; Donnelly et al., 2021; Liedtke, 2021), glia and neurons modulate each other’s activity and, thereby, regulate each other’s phenotypic state (Pinho-Ribeiro et al., 2017). Phenotypic screening assays, wherein functional measurements—not biomolecular abundances—are used to quantify a drug’s relevant effect, are being developed and successfully adopted at increasing rates (Clements and Thomas, 2014; Odawara et al., 2014; Young et al., 2018; Kussauer et al., 2019; Atmaramani et al., 2020). Therefore, it is apparent that drug screening paradigms targeting pathologies of the CNS and/or PNS should not ignore a drug’s acute or chronic phenotypic effects on glia. This is especially true for systems and pathologies with high intra- and inter-cellular complexity, such as in the case of chronic pain (Magni and Ceruti, 2014, 2021; Avraham et al., 2020; Gazerani, 2021). At least three necessary questions emerge, namely: (1) What is the most relevant available model of the neuro-glial functional unit?; (2) What is the most relevant indicator of glial phenotype?; and (3) What is the most direct, high-throughput, and non-invasive way of measuring glial phenotypic activity?

In the case of chronic pain, the primary fundamental neuro-glial unit is the sensory neuron and satellite glial cell(s). Previous studies involving primary rodent sensory cultures, derived from dissected dorsal root ganglion (DRG), have demonstrated the potential for blinded, moderate-throughput drug screening *in vitro* using calcium imaging (Iseppon et al., 2022), axonal outgrowth assays (Atmaramani et al., 2021), or multi-well microelectrode array (MEA) recordings (Middya et al., 2021). However, none of these methods intrinsically measure the contributions of neuroglia, potentially missing or mischaracterizing assay “hits”. Moreover, there are well-established, important inter-species differences between primary rodent and human tissues regarding inflammatory and nociceptive pain pathways (Rostock et al., 2018), making interpretation and translation of drug “hits” challenging. Primary adult human DRG tissue is rare (Valtcheva et al., 2016; Black et al., 2019) and largely inviable for high-throughput culture assays (Enright et al., 2016). Human-derived induced pluripotent stem cells (iPSCs), however, offer a scalable source of human, disease-relevant, cells that can be differentiated into various cell types, including sensory neurons and glial cells (Wainger et al., 2015). While iPSCs are still being studied and optimized for maturation and differentiation, they are becoming widely accepted for addressing phenotypic questions of human disease (Chase et al., 2022a).

It was long held that glia were electrically quiescent (Carmignoto et al., 1998) since they did not obviously contribute to either individual action potential firings or, seemingly, to local field potential recordings. Since 1982 (Kettenmann et al., 1982), however, it has become apparent that glia are not only electrically active, in the form of measurable calcium transients, but that they can directly contribute to neuron-glia-neuron coupling and ectopic neuronal firing *via* gap junctions (Spray et al., 2019). Additionally, the rate and magnitude of glial calcium transients may directly correlate to the cell’s “activity” and may provide additional insight into paracrine (or even autocrine) signaling (Fuchs et al., 2007). Therefore, measuring glial calcium transients may be an appropriate measure for defining glial cells’ phenotypic state. Calcium transients can be measured using calcium indicator dyes (Segond von Banchet et al., 2005) or genetically encoded calcium indicators (GECIs; Grienberger and Konnerth, 2012). However, the usual caveats regarding calcium indicators must be applied; namely, that: (i) these indicators, dyes or GECIs, chelate calcium, potentially affecting intracellular secondary messaging (Wainger et al., 2015); (ii) straightforward imaging approaches are pseudo-quantitative (Sepehri Rad et al., 2017); and (iii) there is potential for photo-toxicity during imaging (Denk et al., 1994). Calcium transients may also be electrically or chemically stimulated and measured using high-sensitivity extracellular MEAs (Fleischer et al., 2015; Mestre et al., 2017a). More specifically, Fleischer et al. (2015) and Theiss et al. (2016) demonstrated the stimulation and measurement of “high-frequency oscillations” or “long-lasting astrocytic potentials” from cultured astrocytes using substrate-integrated MEAs (Mestre et al., 2017a). These studies ascribed high-frequency oscillations (HFOs) to a combination of direct cell-to-cell gap junction communication and glutamate or ATP auto/paracrine signaling. In the intact brain, as well as cortical/hippocampal brain slices, astrocytic HFOs have been observed and are thought to be associated with the development and persistence of bipolar disorder (Stertz, 2021), epilepsy (Pail et al., 2020), and memory formation (Kucewicz et al., 2014; Poskanzer and Yuste, 2016; Szabó et al., 2017). However, to date, no published report has investigated the presence or underlying mechanisms of HFOs in cultures of iPSC-derived glia or, moreover, in a PNS phenotypic model.

Here, we report electrically stimulated OCa^2+^Ts in mono-cultures of iPSC spinal astrocytes as well as co-cultures of iPSC spinal astrocytes and iPSC nociceptors sensory neurons. In both cases, we observed dose-dependent changes in OCa^2+^T magnitude (defined as total power over pseudo-trial baselines) in response to balanced biphasic electrical stimuli with both 800 and 1,200 mV, being applied for phase durations of 200, 400, and 600 μs. These stimulus parameters resulted in OCa^2+^Ts being detected in the immediate vicinity of the stimulated electrodes. We also demonstrated that OCa^2+^Ts can be modulated by the pharmacological glutamate inhibitors AP5/CNQX (75 μM/10 μM) as well as substantially reduced by the gap junction inhibitor, carbenoxolone (CXN, 100 μM), suggesting the necessary role of gap junctions in OCa^2+^Ts. Importantly, both OCa^2+^Ts and single unit extracellular action potentials (EAPs) could be readily distinguished in co-culture recordings, demonstrating a multi-modal approach to determining a drug’s efficacy in blinded electrophysiology-based drug screening paradigms. Lastly, using our workflow, we calculate Z’, an assay quality metric, for electrically evoked OCa^2+^Ts and confirm two mediators of calcium channels and mTOR kinases respectively, Nicardipine (NICA; Young et al., 2018) and Everolimus (EVER; Lang and Borgwardt, 2013), as pharmacological “hits”. In total, these findings suggest that OCa^2+^T stimulation and measurement may be, and perhaps should be, incorporated into future moderate-to-high throughput phenotypic assays aimed at identifying novel analgesics. Additionally, these results suggest a broader applicability to pharmacological assays targeting glia or co-cultures of neuronal and non-neuronal support cells.

## Methods and materials

### Reagents for 2D astrocyte OCa^2+^T electrophysiology

Astrocyte thawing/centrifuging medium: Dulbecco’s Modified Eagle Medium/F12 (Thermo Fisher Scientific, Waltham, MA, USA).

Sensory Neuron Seeding Medium: Custom Senso-MM for co-cultures and Senso-MM for SN monocultures Reagents for seeding and maintaining sensory neuron/astrocyte co-culture were purchased from Anatomic (Minneapolis, MN, USA).

### 48-Well microelectrode array preparation

To give the cells a nutrient rich textured surface to adhere to, 48-well Cytoview MEAs from Axion Biosystems (Atlanta, GA, USA) were prepared for electrophysiological recording and stimulation of iPSC astrocyte and SN cultures by adding a layer-by-layer thin film of polyethyleneimine (PEI) and laminin. The day before seeding, 5 μl of 0.1% (PEI) was placed directly over the electrodes. On the following day, the wells were washed three times with DDI water to remove excess PEI and allowed to air dry. After the wells dried, a 5 μl bead of laminin (20 μg/ml) was placed directly over the PEI area and incubated for 2 h at 37°C in 10% CO_2_. Laminin was aspirated immediately before seeding cells directly over the electrodes.

### Human iPSC seeding

To determine if SNs effect astrocytic HFOs, iPSC astrocytes (BX-0650; BrainXell, Madison, WI, USA) were seeded with and without iPSC SN (Anatomic, Minneapolis, MN, USA). Monocultures and co-cultures of iPSC astrocytes were achieved by seeding and differentiating progenitor cells into astrocytes and SNs on 48 well MEAs (Cytoview, Axion Biosystems, Atlanta, GA, USA). First, the astrocytes and SNs were retrieved from liquid nitrogen and thawed for 30 s in a 37°C water bath. 10 μl of each cell suspension were removed for a cell count while the rest of the cells were transferred to individual 15 ml tubes with 10 ml of cold DMEM/F12 and centrifuged into a pellet at 300 *g* for 5 min. After removing the supernatant, the appropriate amount of Senso-MM was added to each pellet for a cell density of 20,000 astrocytes per 5 μl for monocultures and 20,000 astrocytes plus 50,000 SNs per 5 μl for cocultures and gently triturated until cells were dissociated and suspended. 5 μl of cell suspension was seeded directly over the electrodes of each well, incubated at 37°C and 10% CO_2_ for 30 min until the cells were confluently attached to the bottom, and then the wells were gently flooded with 200 μl of fresh prewarmed Senso-MM cell culture medium. Half medium changes were performed every other day for the life of the cultures.

### Stimulation and recording parameters

To determine the effect of electrical stimulation on astrocytic OCa^2+^Ts, in mono and co-cultures of iPSC astrocytes, electrical stimulation, and recordings were carried out using Axion’s Stimulation Studio Neural Module in combination with Neural Metrics tool and Software Solution provided by Axis 2.3. Mono and co-cultures of iPSC astrocyte and sensory neurons were seeded on a 48-well microelectrode array plate, Cytoview (Axion Biosystem, Atlanta, GA, USA), housing 16 electrodes in each well for a total of 768 electrodes. Baseline recordings were taken every other day from DIV3 to DIV 14 for 30 min, following a 30 min acclimation period in the Maestro system. Continuous data were recorded at 12.5 kHz per electrode and filtered using a 1-pole Butterworth band pass filter from 0.1 Hz to 5 kHz. Once a stable baseline of spontaneous activity was achieved: where we had three consecutive 30 min recordings—each occurring 48 h apart—that exhibited no statistically significant change in either AEY or MFR, we began the stimulation and pharmaceutical experiments. Based on previous experiments monitoring glial calcium transients, our stimulation parameters were set to biphasic, cathodic leading waveforms, with a pulse frequency of 0.05 Hz. On DIV15 baseline electrical stimulation experiments were conducted on both the co-cultures and monocultures. We chose to stimulate the same three corresponding electrodes in each well. We applied, one at a time, six different pulse trains ten times with two amplitudes and four pulse durations consecutively: 800 mV with 200, 400, and 600 μs and 1,200 mV with 400 and 600 μs.

### Data analysis

All electrical stimulation spectrogram data were analyzed in Neural Metric Tools software with a MATLAB script. We calculated the duration and amplitude of evoked OCa^2+^Ts using similar methods as (Fleischer et al., 2015). Briefly, we used a Peri-event Spectrogram analyzer to calculate the TPoB with the time frequency of OCa^2+^Ts from 0 to 600 Hz. We took 30 s of the baseline recording, before stimulation, as the “pseudo-trial” and split the 30 s into six 5 s windows. Within the 5 s windows, an FFT was applied with a Hanning window, and the total power was calculated within each 5 s window as it slid down the signal in 0.5 s increments. The increments were then averaged over the entire 30 s recording for the pseudo trial’s TPoB. To normalize our stimulation data, we subtracted the pseudo trial’s TPoB from the stimulation TPoB and then summed the averaged TPoB for each electrode. Once the TPoB was summed for each electrode we averaged the summed TPoB for each treatment group.

### Pharmacological studies

All compounds were reconstituted in either DMSO or PBS and prepared to have a high, 100× stock concentration in their respective vehicles. This enabled us to add a minute bolus on the far edge of each well to minimize osmolarity changes while the appropriate working concentration was achieved. To evoke OCa^2+^Ts a 5-min baseline electrical stimulation recording of five different stimulus parameters was collected immediately before pharmacological treatments (800 mV for phase duration of 200, 400, 600 μs, and 1,200 mV for phase durations of 400 μs and 600 μs in sequential consecutive order 10 times each with 3 min in between each set). All compounds (NICA, EVER, and CXN) were reconstituted in their recommended solvents (DMSO) at 1,000× working concentration. Following baseline electrical stimulation recordings, cells in four wells were treated with 0.2 μl of 10 mM concentration of each compound or vehicle for 24 h for a total of 16 treated wells. To test the compound mediated responsiveness, after 24 h of incubation, the cells were again subjected to electrical stimulation as described above using an environmental control module in AxIS 2.3.

### Immunocytochemistry

Mono- and co-cultures of iPSC astrocytes and SNs, at DIV 27 and DIV 44, were washed with ice cold PBS three times and fixed with 4% paraformaldehyde for 20 min on a cell rocker. On the day of staining, the cells were first permeabilized with 0.5% Triton X-100 1 h while nonspecific binding sites were blocked with 4% normal goat serum for 2 h. A solution of primary antibodies against glial fibrillary acidic protein (GFAP:1:250; Abcam, Cambridge, MA, USA), calcitonin gene-related peptide (CGRP:1:250; Abcam, Cambridge, MA, USA), s100 beta (s100β: 1:100; Abcam, Cambridge, MA, USA) and Neurofilament 200 (NF200:1:250; Abcam, Cambridge, MA, USA) in PBS and 4% NGS was incubated on the cells overnight. The following day the cells were washed three times with ice cold PBS on the rocker for 10 min each. Then the cells were incubated for 2 h in a solution of secondary antibodies in PBS with of 4% NGS conjugated to their corresponding species with the following wavelengths: 647, 555, 488, and 405 nm. The cells were imaged with a Leica S8 confocal microscope (Leica Biosystems, Danvers, MA, USA). All images were analyzed with ImageJ (NIH, USA).

## Results

### Immunocytochemistry

**Figure 1:**
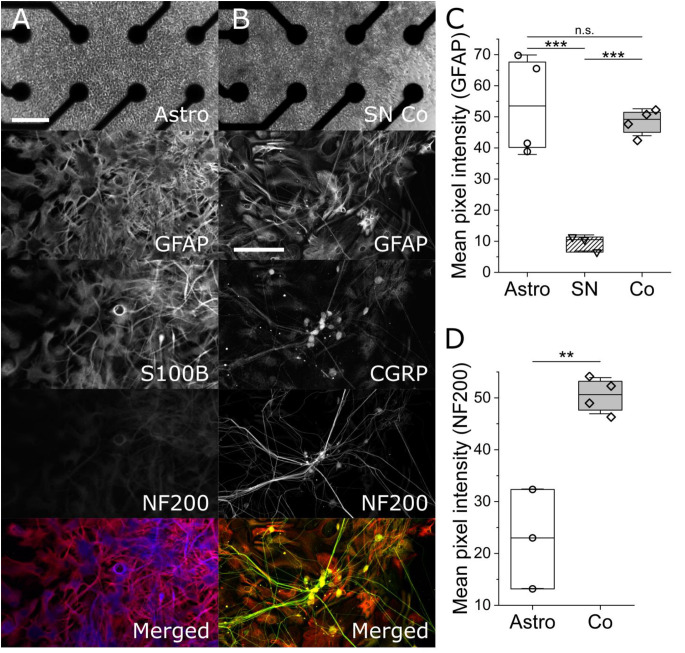
Phase-contrast and maximum-intensity projection immunofluorescent images of mono-culture iPSC astrocytes (column **A**) and iPSC sensory co-cultures (column **B**). Column **(A)** shows a phase contrast image of monoculture iPSC astrocytes on an Axion multi-well Cytoview MEA. The scale bar represents 100 μm. ICC images show glial fibrillary acidic protein (GFAP, red), S100β (blue), neurofilament 200 kDa (NF200, green), and a merged image. Column **(B)** shows (GFAP, red), calcitonin gene-related peptide (CGRP, yellow), and marker for peptidergic sensory neurons, Neurofilament 200 kDa (NF200, green. The associated scale bar indicates 150 μm. Panels **(C)** and **(D)** show the quantification of GFAP- and NF200-associated mean pixel intensities, respectively. **, *** indicates *P* < 0.01, and 0.001. n.s. indicates no significant difference.

To determine the purity of iPSC astrocyte and heterogeneity of SN co-cultures, we performed ICC on parallel cultures on similarly prepared glass-bottom multi-well plates. The top panel of Figures 1A,B are phase contrast images of an hiPSC astrocyte mono-culture on the left and an hiPSC co-culture of astrocytes and neurons on the right, atop of electrodes in an Axion Biosystems’ Cytoview microelectrode array. The scale bar is 100 μm. The second panel in column Figure 1A shows the expression of GFAP and S100β, two markers for astrocytes and mature astrocytes, respectively. Importantly, we observed statistically similar GFAP-associated pixel intensities across astrocyte mono- and co-culture conditions (Figure 1C, 53.89 ± 13.84 vs. 48.26 ± 3.75, *P* = 0.52, two-sample *t*-test), but statistically lower GFAP intensity in mono-cultures of sensory neurons only (9.46 ± 2.11, *P* = 0.005, two-sample *t*-test). In Figure 1B, sensory co-cultures show distinct non-overlapping markers for astrocytes, GFAP, and neurons, NF200 and CGRP, and, further, observed a 1:1 overlap of CGRP, a marker of peptidergic afferent sensory neurons, with that of NF200. Importantly, we observed significantly higher NF200-associated pixel intensity in mono-cultured astrocytes vs. co-cultures of spinal astrocytes and sensory neurons (Figure 1D, 50.42 ± 3.01 vs. 22.83 ± 7.83, *P* = 0.0028, two-sample *t*-test), which is supported by our MEA astrocyte recordings; exhibiting no measured action potentials over more than 30 days *in vitro* (data not shown) as well as vendor (BrainXell, Inc) guarantees of astrocytic purities greater than 90%. This suggests that neither the combined maintenance medium, nor the presence of the other cell type, leads to dedifferentiation or common, but aberrant, differentiation.

### Electrical stimulation and evoked activity

**Figure 2:**
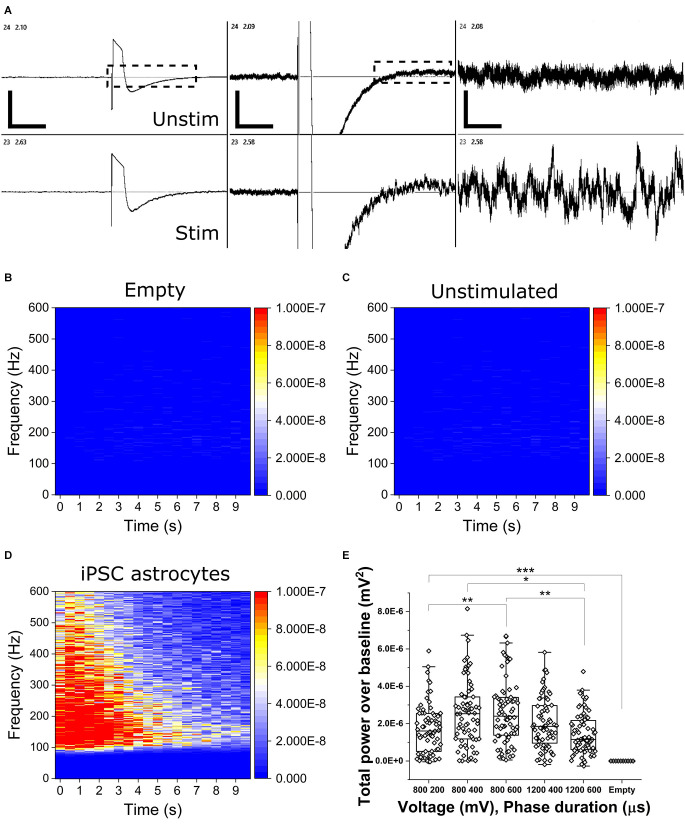
Electrically stimulated OCaTs in monocultures of iPSC astrocytes. **(A)** Representative continuous traces of voltage vs. time data for two adjacent electrodes, one unstimulated (top, unstim) and one stimulated (bottom, stim). From left, the scale bars represent, 5, 2, and 1 s (horizontal) and 650, 125, and 25 μV (vertical). Electrodes are spaced 350 μm apart. **(B,C,D)** Heat maps of TPoB from a stimulated electrode in a well with only culture medium **(B)**, an unstimulated electrode in a well with astrocytes **(C)**, and a stimulated electrode in a well containing a monoculture of astrocytes **(D)**. Panel **(E)** shows TPoB for each stimulated electrode (hollow diamonds) in a monoculture of astrocytes for five different stimulus parameters. Boxes represent the 25th and 75th percentile with median lines and Standard Deviation whiskers. Statistical comparison: * indicates *p* ≤ 0.01, ** indicates *p* ≤ 0.001, and *** indicates *p* < 0.001, respectively.

To determine the presence of OCa^2+^Ts in populations of mono-cultured iPSC astrocytes, we applied bi-phasic, charge-balanced, cathodic-leading electrical pulses of varying voltage-controlled amplitudes (800 and 1,200 mV) and phase durations (200, 400, and 600 μs). Figure 2A shows representative continuous voltage traces depicting voltage vs. time from a stimulated electrode (bottom trace, 800 mV, 600 μs) and an adjacent unstimulated electrode (top trace). OCa^2+^Ts could be readily observed in real time, persisting for up to 20 s. OCa^2+^T amplitudes are reported as total power over baseline (TPoB) as compared with each electrode’s pseudo-trial average, as described in the Methods section. Figures 2B–D show heat maps of average TPoB vs. frequency vs. time for stimulus applied to an empty well containing culture medium (Figure 2B), an unstimulated well containing an astrocyte mono-culture (Figure 2C), and one which was both seeded and stimulated (Figure 2D). The TPoB significantly increased in a dose-dependent manner, from the application of 800 mV for 200 μs triggering a TPoB amplitude of (1.7E^−6^ ± 1.32E^−6^) to 800 mV for 600 μs eliciting an average amplitude of (2.62E^−6^ ± 1.67E^−6^ mV^2^, *X*^2^_(12.7)_ = 144, Kruskal-Wallis ANOVA, *p* = 0.014). TPoB then decreased significantly with the application of 1,200 mV for 600 μs to (1.46E^−6^ ± 1.11E^−6^ mV^2^, *X*^2^_(13)_ = 144, Kruskal-Wallis ANOVA, *p* = 0.0003). All stimulus parameters resulted in significantly higher TPoB vs. stimulation applied to electrodes in an unseeded well (−1.17E^−9^ ± 9.28E^−10^ mV^2^, *X*^2^_(51)_ = 370, Kruskal-Wallis ANOVA, *P* < 0.0001), thus illustrating little-to-no contribution of stimulation or biological artifact.

### Preliminary drug screening

**Figure 3:**
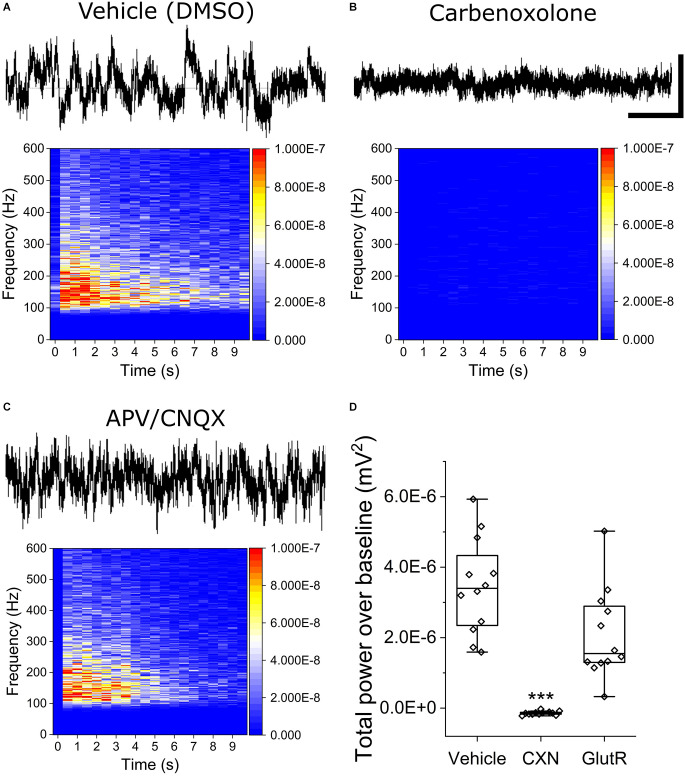
Stimulated OCaTs from iPSC astrocyte cultures are modulated by gap junction inhibitor, carbenoxolone, but not by glutamate receptor antagonists. **(A–C)** Representative continuous traces of voltage vs. time data for DMSO (Vehicle, top left), carbenoxolone (top right), and a combination of glutamate receptor antagonists (APV/CNQX, bottom left). The scale bar represents 5 s (horizontal) and 50 mV (vertical). **(D)** Statistical comparison of the three treatment groups. *** indicates *p* < 0.001. The box lines are median, the edges are 25th and 75th percentile, and the whiskers are outliers with coefficient 1.5.

To determine the mechanistic basis of OCa^2+^Ts in iPSC astrocyte mono-cultures, we repeated stimulation experiments using the optimally effective baseline stimulation parameters of 800 mV amplitude and 600 μs phase duration in the presence of gap junction and glutamate receptor antagonists. Stimuli were applied approximately 2 min following the addition of either 100 μM CXN, a gap junction inhibitor, or a combination of 75/10 μM AP5/CNQX, potent and selective glutamate receptor (GlutR, NMDA, and AMPA) antagonists, respectively. Figures 3A–C show raw continuous voltage traces and heat maps for the vehicle (0.1% DMSO), CXN, and GlutR antagonists. Figure 3D is a box plot for statistical comparison of TPoB for each stimulated electrode (hollow diamonds) in each treatment group. We observed a significant decrease in TPoB after the addition of CXN (−1.43E^−7^ ± 4.6E^−8^ mV^2^, as compared to electrodes in vehicle-treated wells (3.46E^−6^ ± 1.69E^−6^ mV^2^, *X*^2^_(36)_ = 26, Kruskal-Wallis ANOVA, *p* = 0.002). However, the glutamate receptor antagonists, AP5/CNQX (2.08E^−6^ ± 1.13E^−6^ mV^2^, *p* = 0.19) did not induce a significant decrease in TPoB as compared to the vehicle wells (*X*^2^_(36)_ = 26, Kruskal-Wallis ANOVA, *p* = 0.26).

**Figure 4:**
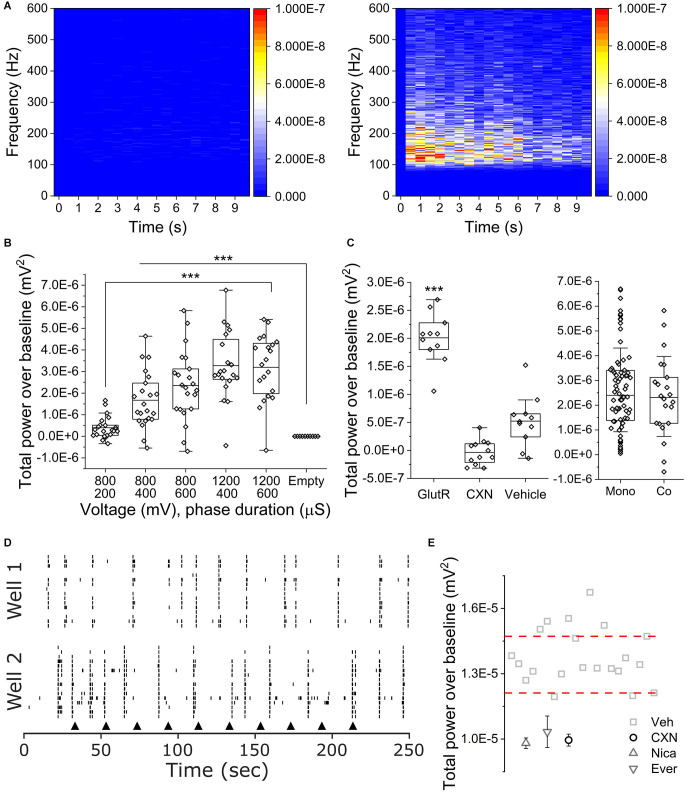
OCaTs in iPSC SN and astrocyte co-cultures. **(A)** Frequency- and time-dependent heat maps of averaged TPoB for an empty (left) and co-cultured (right) wells following 10x repetitions of 1,200 mV, 400 μs phase duration stimuli. **(B)** Voltage- and phase duration-dependence of co-culture OCaTs. The lines are mean, the edges are 25th and 75th percentile, and the whiskers are outliers with coefficient 1.5. **(C)** TPoB subsequent to APV/CNQX (GlutR), carbenoxolone (CXN), or DMSO (VEH) treatments and statistical comparison to baseline TPoB for 800 mV, 600 μs phase duration stimuli. The lines are mean, the edges are 25th and 75th percentile, and the whiskers are outliers with coefficient 1.5. ***indicates *p* < 0.001, respectively. Comparison of TPoB for 800 mV, 600 μs phase duration stimuli between mono- and co-cultures. The lines are median, the edges are 25th and 75th percentile, and the whiskers are SD with coefficient 1. **(D)** Representative raster plots of single-unit activity recorded from separate sensory neuron co-cultures subject to repeated electrical stimulation (black arrowheads) with 800 mV, 600 μs phase duration. **(E)** TPoB after 1,200 mV for a phase duration of 400 μs was delivered to electrodes in cocultures wells with pharmacological compounds NICA, EVER, and CXN. Thresholds (dashed lines) indicate pharmacological “hits.”

To determine if co-cultures of iPSC SN and astrocytes exhibited OCa^2+^Ts, we seeded co-cultures in 48 well Cytoview MEAs. We applied five different stimulus parameters in consecutive order as we did with the monocultures. Figure 4A shows peri-event spectrograms from 100 to 600 Hz for 10 s of TPoB OCa^2+^Ts activity from an empty well on the left with an amplitude of (−1.17E^−9^ ± 9.28E^−10^ mV^2^) and a cultured well on the right with an amplitude of (3.19 E 10^–6^ ± 1.49 E 10^–6^ mV^2^) after delivering 1,200 mV for a phase duration of 600 μs. Figure 4B is a box plot showing the averaged TPoB amplitude for each stimulated electrode (hollow diamonds) for each stimulus parameter in a co-culture and an empty well containing only cell culture medium. The TPoB in the co-cultures increased significantly in a dose dependent manner from applying the stimulus parameters: 800 mV for a phase duration of 400 μs, to 1,200 mV for a phase duration of 600 μs (1.67E^−6^ mV^2^ ± 1.2E^−6^ to 3.19E^−6^ mV^2^ ± 1.49E^−6^, *X*^2^_(57)_ = 116, Kruskal-Wallis ANOVA, *P* < 0.0001). All stimulus parameters applied in the cultured wells showed a significantly higher TPoB OCa^2+^T amplitude than in the empty wells (−1.17E^−9^ mV^2^ ± 9.27E^−10^, *X*^2^_(57)_ = 116, Kruskal-Wallis ANOVA, *P* < 0.0001) Next, in Figure 4C, we show the average TPoB for each stimulated electrode after pharmacological treatments and a comparison between mono- and co-cultures exhibiting similar TPoB averages after 800 mV for a phase duration of 600 μs. We treated four wells each with either 100 μM CXN, 75/10 μM AP5/CNQX, or 0.1% vehicle (DMSO) 2 min prior to applying 800 mV for a phase duration of 600 μs. The GlutR-treated wells exhibited a TPoB of OCa^2+^T amplitude that was significantly higher than the vehicle treated wells (2.01E^−6^ ± 4.23E^−7^ and 5.24E^−7^ ± 4.32E^−7^ mV^2^, *X*^2^_(22)_ = 15.3, Kruskal-Wallis ANOVA, *P* < 0.01), while the TPoB amplitude in the CXN treated wells was not significantly lower than the TPoB amplitude recorded from electrodes in vehicle treated wells (−3.51E^−8^ ± 2.1E^−7^ mV^2^, CXN vs. VEH; *X*^2^_(22)_ = 9.8, Kruskal-Wallis ANOVA, *P* = 0.09, Figure 4C). There was not a significant difference in TPoB amplitude between the mono- and co-cultures using the stimulus parameters 800 mV, 600 μs (*X*^2^_(94)_ = 3.2 Kruskal-Wallis ANOVA, *P* = 0.6). Figure 4D is representative of raster plots of single unit activity recorded from separate hiPSC sensory neuron co-cultures subjected to repeated current injection of 800 mV for a phase duration of 600 μs (black arrows). Importantly, the electrical stimulation did not significantly affect extracellular action potential firing. Peri-event histogram analysis immediately before and after electrical stimulation (± 0.5 s) indicated neither a significant difference in the probability of recording an extracellular action potential (23.1 vs. 22.9%, *p* = 0.944, *n* = 482, two-sample test of proportions) or the mean number of extracellular action potentials recorded in those periods (1.32 ± 1.27 vs. 1.21 ± 1.06, *p* = 0.35, *n* = 482, Mann-Whitney U-test).

### Assay quality Z’ metric and preliminary “hit”

To assess the quality of our OCa^2+^T model as a compound hit assay affecting glia, we calculated its Z’ score, which is mathematically defined as *Z’* = 1-[(3σc^+^+ 3σc^−^)/|μc^+^ – μc^−^|], and is a cell-based assay quality metric, as mathematically described previously (Zhang et al., 1999). We used the stimulus parameters, 1,200 mV for a phase duration of 400 μs, to achieve a calcium response for our positive control and the gap junction inhibitor CXN (100 μM) to reduce calcium transients, for our negative control. Because CXN reduced amplitudes such that some TPoB calculations were small negative values, we added 1E-9 in order to make all TPoBs positive for the purpose of log normalization. For consistency across controls, we did the same to the positive control data. With these parameters we achieved a Z’ of 0.55 with a signal window of 4.88 and an assay variability ratio of 0.45 which is considered “recommended” and “an excellent assay” by the previous articles (Zhang et al., 1999; Iversen et al., 2006; Todd Kuenstner et al., 2019; Atmaramani et al., 2020). In brief, these numbers suggest that the protocols detailed here are sufficient as a stand-alone analytical tool for glial phenotype.

Figure 4E shows the log TPoB/electrode across all active wells from a single plate, along with ±3 times the median absolute deviation threshold lines. Compounds below the thresholds may be considered as hits for inhibiting calcium transient activity. To determine if our assay could identify cherry-picked FDA approved compounds from different classes of analgesics, we applied CXN (10 μM), NICA (10 μM), EVER (10 μM), and vehicle (DMSO) to four wells per compound. Each compound scored (−3.4E-8 ± 2.11E-7: CXN), (−1.5E-7 ± 1.7E-7: NICA), and (2.7E-7 ± 5.9E-7: EVER), far below the bottom threshold of the positive control. All three compounds scored as a hit, suggesting our assay can identify compounds that act on OCa^2+^Ts.

## Discussion

Along with EAP and local field potential (LFP) recordings, electrophysiological activity in the “high-frequency oscillation” range (100–600 Hz) has long been associated with central nervous system function (e.g., memory formation (Kucewicz et al., 2014), and changes in these oscillatory patterns have been associated with pathological dysfunction (Buzsáki and Lopes da Silva, 2012). However, *in vivo* HFOs have long been considered a neural phenomenon (Li et al., 2021). Only in the past 25–30 years has the electrophysiological contributions of glial cells been even considered (Berger et al., 1991), with their fundamental importance being recognized much more recently than that (Temburni and Jacob, 2001). It is now an open question whether certain pathologies of the central or peripheral nervous system are, in fact, gliopathies (Ji et al., 2013, 2019; Magni and Ceruti, 2021). Regardless, it is now beyond question that glia play a critical role in central and peripheral nervous system disorders (Gwak et al., 2012; Cirillo and Papa, 2016; Pochard et al., 2018; Ji et al., 2019; Stertz, 2021; Sheridan et al., 2022) and that they are a viable therapeutic target that should be addressed in drug discovery and development (Assefa et al., 2018; Garcia et al., 2019; Vanderwall and Milligan, 2019; Ceruti, 2021). Thus, the need to develop compound hit assay to investigate glia functional responses.

Contemporary drug discovery is increasingly targeting phenotypic activity by measuring functional outcomes from relevant cell populations. Moderate-to-high throughput phenotypic screening which addresses neuroglial activity is predominantly carried out using either multi-well calcium imaging (Iseppon et al., 2022) or MEA platforms (Fleischer et al., 2015; Moy et al., 2017; Black et al., 2018; Atmaramani et al., 2020; Chase et al., 2022b). While efforts have been made to integrate both methods into a single platform (Middya et al., 2021), a number of key challenges remain in multi-well configurations which we will not explore here. Suffice it to say that both methods have prospective advantages and disadvantages. In the case of calcium imaging, one of the important disadvantages has been the distinction of neural and glial cell types which may also exhibit calcium transients. This challenge is being addressed in two ways: (1) the advent of genetically-encoded calcium indicators enables measurements from individual cell types (Sepehri Rad et al., 2017). However, the incorporation of multiple promoters and fluorescent proteins into a single culture such that multiple classes of cells can be simultaneously imaged has not been demonstrated to the best of our knowledge. Therefore, the challenge of discriminating cell types is outstanding. (2) Cell identity can be discerned following a series of agonist/antagonist treatments paired with size and circularity measurements in a semi-automated way (Atmaramani et al., 2021). However, to measure calcium transients in both neuronal and glial populations either requires multiple GECI probes or uses an acetoxymethyl (AM) ester uptake mechanism to deliver dyes with known long-term toxicity (e.g., Fluo-4AM; Tcw et al., 2017). Non-invasive calcium transient detection, as described here, therefore presents a unique opportunity to detect drug hits or leads that may act on either neurons or their associated glia. To date, and to the best of our knowledge, there have been only two published reports which demonstrate electrically stimulated OCa^2+^Ts (or “HFOs”) *in vitro*. Table 1 summarizes these studies. While there are subtle differences in the stimulation parameters and pharmacological perturbations, the three are largely consistent in mechanistic observations. For instance, blocking GlutRs does not significantly attenuate OCa^2+^Ts and that CXN (a gap junction inhibitor) does significantly attenuate OCa^2+^Ts. Despite this agreement, future work is required to correlate the magnitude and duration of calcium transients with OCa^2+^Ts. While previous studies have reported simultaneous electrical stimulation and calcium imaging (Mestre et al., 2017b) none have simultaneously recorded OCa^2+^Ts using MEAs to date.

**Table 1:** Comparison of previously published cell-based assay acceptance criteria and that of our iPSC SN and astrocyte co-culture model’s TPoB-based scores.

Acceptance Criteria for Signal Window, Z’, Assay Variability Ratio		
Signal Window (SW)	Assay Variability Ratio (AVR)	Z’_M_
Recommended: SW > 2 Acceptable: SW > 1 Unacceptable: SW < 1	Recommended: AVR < 0.6 Unacceptable: AVR > 0.6	Recommended: Z’ > 0.4 Excellent 0.5 ≤ Z’ ≤ 1 useable 0 < *Z’* < 0.5
Our iPSC- based assay scores: positive control/negative control		
EStim/CXN: SW = 4.88	EStim/CXN: AVR = 0.45	EStim/CXN Z’_M_ = 0.55

Here, we report the first simultaneous quantification of electrically-evoked iPSC astrocytic OCa^2+^Ts and iPSC sensory neuron action potentials using multi-well MEAs. OCa^2+^Ts in mono- and co-cultures of iPSCs exhibited durations of up to 20 s with the highest TPoB in the 100–200 Hz frequency range (Figure 2). Importantly, at stimulation parameters most effective in the mono-culture (800 mV, 600 μs), there was no statistically significant difference in OCaT amplitudes between mono- and co-cultures, further suggesting that the presence of sensory neurons did not substantially affect OCaT amplitudes. These findings are largely in line with previous studies in regard to amplitude and duration (though differently defined), as summarized in Table 2. iPSC astrocyte mono-culture OCa^2+^Ts did not exhibit a biomodal “dose” dependency above 800 mV and 600 μs, suggesting a possible depletion of either intracellular calcium or other mediators of intercellular communication [e.g., IP3 (Lia et al., 2021)]. Additionally, we validated the previously proposed mechanistic basis for OCa^2+^Ts; measuring statistically significant decrease in the presence of gap junction inhibitor CXN (Figure 3). Notably, glutamine receptor antagonists AP5/CNQX did not significantly modulate OCa^2+^Ts in mono-cultures but did so in our co-cultures (Figures 3 and 4). Previous studies (Perea et al., 2009) have suggested additional mechanistic contributions involving the activation of G_q_ GPCRs thereby stimulating the production of phospholipase C and IP3, which were not specifically tested here. This fact, combined with the relative complexity of neuroglial interactions, would open the door to the identification of other contributing factors yet to be elucidated. However, it is worth noting that CXN effectively suppressed OCa^2+^Ts, suggesting a primary, sufficient role.

**Table 2:** Comparison of presently reported (p.s.) outcomes and those of previous MEA-based calcium transient measurements.

Ref	Cells	Stimulus (amp, ph duration)	Amplitude	Duration	Mechanistic observations
Hales et al. (2012)	E18 rat hippocampus and cortical co-cultures	300–700 mV, 400 μs	16–25 μV	0.7–2.9 s	TTX resistant, CXN responsive, APV/CNQX resistant
Fleischer et al. (2015)	P0 Wistar rat cortical astrocytes or co-cultures	800 mV, 200 μs	1–5 × 10^–9^ V^2^	2.4 s	TTX resistant, APV/CNQX resistant, clinidipine responsive, CXN responsive, Bafilomycin responsive
p.s.	iPSC spinal astrocytes and iPSC sensory co-cultures	800–1,200 mV, 200–600 μs	0.5–6 × 10^–6^ mV^2^	2–20 s	APV/CNQX resistant, CXN responsive

In order to move from preliminary demonstration to relevant pre-clinical assay, we calculated the Z’ score (Table 2), a widely used assay quality metric for moderate-to-high throughput phenotypic assays. As detailed in the associated results section, we observed a Z’_M_ of 0.55 with a signal window of 4.88 and an assay variability ratio of 0.45 which is considered “recommended” and “an excellent assay” by previous studies (Zhang et al., 1999; Iversen et al., 2006; Todd Kuenstner et al., 2019; Atmaramani et al., 2020). We then screened two FDA-approved compounds known to effect astrocyte-expressed calcium or voltage gated calcium channels. Both compounds were identified as “hits” in the iPSC sensory co-culture (Figure 4), demonstrating the integrated potential of dual screening of sensory neurons and astrocytes.

It has long been understood that gliotransmitters are calcium dependent and that these transmitters modulate neuronal activity (Ahmadpour et al., 2021). However, it is still unclear how and to what extent reactive astrocytes regulate or contribute to malsynaptic plasticity and or noxious nociceptive molecular mechanisms. There is mounting evidence that suggests astrocytic gliopathy may lead to neurological disorder, including chronic pain states (Gwak et al., 2012; Kenyon et al., 2015; Cirillo and Papa, 2016; Pochard et al., 2018; Zimmer et al., 2020; Magni and Ceruti, 2021; Stertz, 2021). Therefore, when studying pain pathologies *in vitro*, it is paramount to include and monitor non-neuronal support cells. Previous studies using iPSC SN that have been co-cultured with support cells have used only extracted rodent Schwann cells (Cai et al., 2017), or rodent astrocytes (Odawara et al., 2014) for the purpose of studying nociception. To the best of our knowledge, this is the first nociceptive phenotypic screening platform using human iPSC sensory neurons together with iPSC astrocytes. Importantly, we show that astrocytes can be monitored, alongside SNs, in real time, non-invasively for the life of the culture. By monitoring astrocytic calcium transients with MEAs, we can see the effects of potential therapeutic compounds on both cell types, and our findings are in line with previous studies of astrocytic calcium transients (Fleischer et al., 2015).

## Data availability statement

The raw data supporting the conclusions of this article will be made available by the authors, without undue reservation.

## Author contributions

JL and BB: conceptualization. JL, EL, and BB: methodology. EL, MD, JL: formal analysis. BB: resources, supervision, and funding acquisition. JL, EL, MD, and BB: writing—original draft preparation, writing—review and editing. All authors contributed to the article and approved the submitted version.
